# Screening and Referral for Postpartum Depression among Low-Income Women: A Qualitative Perspective from Community Health Workers

**DOI:** 10.1155/2011/320605

**Published:** 2011-04-28

**Authors:** Rhonda C. Boyd, Marjie Mogul, Deena Newman, James C. Coyne

**Affiliations:** ^1^Department of Child and Adolescent Psychiatry and Behavioral Science, Children's Hospital of Philadelphia and University of Pennsylvania School of Medicine, 3535 Market Street, Suite 1230, Philadelphia, PA 19104, USA; ^2^Research Department, Maternity Care Coalition, 2000 Hamilton Street, Suite 205, Philadelphia, PA 19130, USA; ^3^Department of Psychology, La Salle University, 1900 West Olney Avenue, Philadelphia, PA 19141, USA; ^4^Department of Psychiatry, University of Pennsylvania School of Medicine, 3535 Market Street, 6th Floor, Philadelphia, PA 19104, USA

## Abstract

Postpartum depression is a serious and common psychiatric illness. Mothers living in poverty are more likely to be depressed and have greater barriers to accessing treatment than the general population. Mental health utilization is particularly limited for women with postpartum depression and low-income, minority women. As part of an academic-community partnership, focus groups were utilized to examine staff practices, barriers, and facilitators in mental health referrals for women with depression within a community nonprofit agency serving low-income pregnant and postpartum women. The focus groups were analyzed through content analyses and NVIVO-8. Three focus groups with 16 community health workers were conducted. Six themes were identified: (1) screening and referral, (2) facilitators to referral, (3) barriers to referral, (4) culture and language, (5) life events, and (6) support. The study identified several barriers and facilitators for referring postpartum women with depression to mental health services.

## 1. Introduction

Postpartum depression (PPD) is a serious and common psychiatric disorder that can have negative effects on women, their children, and families. Among chronic diseases, depression places the second greatest burden on women's health worldwide [1]. Research has estimated that approximately 6.5%–13% of women develop depressive symptoms within the first postpartum year [2, 3]. There is consistent evidence indicating that women who have experienced PPD are at an increased risk for future episodes of depression than women who have not experienced PPD [4–6]. Women living in poverty generally show higher rates of PPD than women who are more financially affluent [7–9]. It is estimated that approximately 13.2% of the United States population lives below the poverty level [10]. Economically disadvantaged women are particularly vulnerable since they may experience challenges in having their depression detected [11]. 

The implementation of widespread screening for depression in postpartum women has been recommended [12, 13]. Although some have argued that screening for depression is problematic and at times unwarranted [14], others point to the benefits of screening, including identifying those in need of mental health services, detecting depression in underserved populations, and preventing mental health problems in mothers and their children [15, 16]. A recent study of the Healthy Start Depression Initiative showed that universal perinatal depression screening did not increase depression treatment and may even have decreased the rate of referral for services [17]. There is a need to address barriers in the referral process if postpartum mothers are to receive adequate care for their depression.

There has been only limited research focusing on mental health service utilization during the postpartum period. In the Song et al. [18] study examining Medicaid records of pregnant and postpartum women in a large urban city, 6.4% of the women utilized mental health services within six months postpartum, all of whom attended at least one outpatient visit. Approximately 13% of the postpartum women who utilized mental health treatment were diagnosed with Major Depressive Disorder (MDD). In a large community screening study of PPD, only 12% of the women screening positive received psychotherapy and between 3.4–6% received medication at three and four months postpartum [19]. A national survey of PPD symptoms showed that 57% of women with moderate to severe symptoms did not seek help from health or mental health providers [8]. These latter two studies indicate low mental health utilization in a community sample. 

General barriers for women with PPD include shame, feelings of failure due to having PPD, childcare, family responsibilities, lack of knowledge about where to seek treatment and treatment options, lack of knowledge about PPD, stigma, doubt about treatment effectiveness and perceived insufficient time [20, 21]. A qualitative study of emotionally distressed low-income mothers who had a child in mental health treatment demonstrated that barriers for the mothers' own mental health utilization also include a lack of identification with a depression diagnosis, external attribution about the causes of depressive symptoms, and the belief that a child's needs should come before her own [22]. Some have noted that the link between detection and improved outcomes is mediated by access to services [19, 23]. However, postpartum women living in poverty are likely to have greater barriers to accessing mental health services [18].

To address PPD for low-income women, we have an existing academic-community partnership which has focused on screening and barriers to mental health utilization in a large urban city. Community health workers play a major role in provision in care for postpartum women in this partnership. Community health workers are trained paraprofessionals; most of whom are either from or reflect the communities which they serve. They conduct home visits to pregnant and postpartum women providing education, social support, access to benefits and assistance navigating the healthcare delivery system. They also screen for PPD and assist in referring women to mental health services. Other studies have demonstrated the effectiveness of community health workers in the provision of services for PPD [24, 25]; however, limited information is available from the community health worker's perspective. As a result, we conducted focus groups with community health workers in a community-based organization in order to gain understanding of the screening process and barriers and successes in mental health referrals for women with depression. In this paper, we present the findings of the focus groups and discuss the strategies for screening and assisting low-income women with PPD to initiate mental health services.

## 2. Materials and Method

### 2.1. Sample Characteristics

Sixteen female community health workers participated in three focus groups. The number of participants in the groups ranged from 3 to 8. The number of years that the participants worked at this agency ranged from less than a year to fifteen years, with a mean of 2.3 (SD = 1.08). These participants represent 57.1% (*n* = 16) of the community health workers employed at the agency. The sample characteristics of the participants are presented in Table 1.

### 2.2. Agency

The community-based partner is a private nonprofit organization founded to address infant mortality and morbidity in a large urban setting. The agency provides direct service, research and advocacy in high-risk neighborhoods through the use of a home visiting model using community health workers. Approximately 2600 women receive services annually by the community health workers. The agency defines low-income as the receipt of public benefits. The agency has a perinatal depression program which screens its clients for depression and provides supportive and preventative services. This perinatal depression program began approximately eight months prior to the focus groups. At the time of the current study, a masters level clinician conducted clinical interviews with women who screened high for depressive symptoms. Mental health treatment is not provided by the agency. As a result, if a woman is in need of mental health services, then the masters level clinician and/or community health worker would assist her in seeking care. 

### 2.3. Recruitment

At an agency staff meeting, we presented an overview of the research program and focus group procedures. The goal was to recruit community health workers to participate in the focus groups. A followup email was sent to all community health workers inviting them to attend focus groups. Community health workers signed up for selected dates and could only participate in one focus group.

### 2.4. Procedures

Focus groups were conducted at the main office of the agency from April 2008 through May 2008. Each focus group lasted approximately 90 minutes and was audio taped. The second author moderated the focus groups and collected verbal consent at the beginning of each group. The study was approved by the Institutional Review Board of the Children's Hospital of Philadelphia and was conducted in Philadelphia, Pennsylvania in the United States.

Several topical questions guided the discussion in each focus group. Focus group topics and their corresponding questions were developed jointly by the first three authors to gather information concerning the community health workers' knowledge and practices in screening for PPD and referring women to mental health services. Structured, open-ended questions with guided prompts were developed, based on existing literature and agency experience. The focus groups were held in a manner in which participants were encouraged to discuss new ideas about mental health referral for women with PPD [26]. The core topics for the focus group interviews were (1) referral efforts, (2) frequency of referrals, (3) referral sources, (4) barriers to referral, (5) referral strategies perceived to be effective, and (6) followup procedures to ensure referrals have been completed. 

### 2.5. Data Analyses

The audiotape of each focus group was transcribed and the transcripts were then checked against the audiotapes. The third author wrote notes during each focus group interview which were also used as needed in the transcription process. A multiphased process was used to analyze the transcribed interview data based on content analytic methods [27]. Three coders, which included the authors, coded each transcript independently with open coding of units of analysis. Next, they identified initial categories and engaged in comparative analysis. All three coders were in agreement for initial categories to be identified and recorded. The content analyses were performed with NVIVO-8. Through comparative analyses and an iterative process, major themes were identified by consensus between the primary and secondary authors. Finally, the initial sets of categories were reorganized around the major themes.

## 3. Results

The focus groups identified six major themes (1) screening and referral, (2) facilitators to referral, (3) barriers to referral, (4) culture and language, (5) life events, and (6) support. 

### 3.1. Screening and Referral

This theme encompasses the screening and referral process, as well as training in depression screening and mental health referral. The community health workers described their depression screening in which their clients are referred to the agency's perinatal depression program if they score above a clinical cut-off on the Edinburgh Postnatal Depression Scale (EPDS, [28]), a self-report measure of postpartum depression symptoms. The workers also noted difficulties with screening that included both an overidentification and underidentification of depression. One community health worker stated “I think that the screening tool, because…it measures (depression) within the (past) seven days…gives…(a) false number, and…makes it seems like…they are more elevated or/and they are more in need of a dire intervention because of how the questions are asked…”.

In regard to referrals, some community health workers give a client the telephone number of the referral agency directly and other workers will call the referral agency themselves. Numerous phone calls and a large amount of paperwork are required to complete the screening and referral process.

 The workers review a long list of referral agencies to determine which are appropriate and geographically convenient for each client. Finding the proper approach to make a referral becomes a challenge as one community health worker said:

“Every client is different,…Sometimes I just clump them all together and everybody gets the same treatment…Where ever they are, you have to meet them there and try to help them rise up from it…”

Most community health workers described several trainings on both PPD and the administration of the EPDS. Some workers felt trained and capable for mental health referral; however, others, especially those new to the agency or the work, desired more training. Training needs included making a mental health referral, introducing PPD to a client, and knowledge about psychiatric medications. 

### 3.2. Facilitators to Referral

The community health workers identified facilitators they used to successfully engage women with PPD into mental health services. These facilitators were mostly based on relationships with either the client or staff at mental health agencies. Some community health workers assisted their clients in making the first call to arrange an initial mental health appointment. They also noted that they would accompany clients to their initial appointment at a mental health clinic or help to arrange escorts. If the community health workers were not available, they would arrange for transportation for their client. In addition, many of the community health workers have a contact person at different mental health agencies whom they can contact directly. One community health worker explained that this agency contact person “can expedite your call or give you information that will be beneficial for your client.” Community health workers sometimes get contacts through their peers to facilitate referrals and rely on this informal exchange of information to help refer clients to the appropriate mental health services. One community health worker summed up how she facilitates referrals:

“…Sometimes…you use a first name, give a description of the office, (and) push the relationship…You are there while (the clients) make the phone call and you talk to (the agency) and…say, “here she is right now”…Making a partnership in trying to get this referral to the client and if you can, their accompaniment during the first appointment.”

### 3.3. Barriers to Referral

The community health workers identified numerous barriers to referring postpartum mothers with depression to mental health services. These barriers fall into five categories: (1) practical barriers, (2) personal barriers, (3) stigma, (4) mental health system barriers, and (5) internal agency barriers. The practical barriers include logistical impediments for mothers in attending mental health appointments, such as lack of transportation, lack of childcare, difficulty paying for mental health care, denial about health care and health insurance limitations (e.g., limited number of mental health sessions allotted). One community health worker commented on the childcare barrier, “they cannot take their child with them to their session…(and) a lot of times they cannot afford day care.” The personal barriers described were a lack of motivation to attend mental health services, mistrust, fear of mental health service systems, failure to follow up with appointments and low priority for mental health. In addition, workers reported that other psychiatric disorders, such as substance disorders and bipolar disorder, make it difficult to refer postpartum women to mental health services. 

Stigma regarding psychiatric disorders including PPD was noted as a barrier to referral for services. Community mental health workers perceived the screening process for PPD which identify women who score above the clinical cut-off as stigmatizing clients by labeling them with a psychiatric disorder. One community health worker stated “some (women) are afraid of the stigma that the general…public have on mental health; the public…does not embrace mental health as a real issue.”

Mental health system barriers described were a lack of services, such as mobile and crises units. However, some community health workers agreed that there are an adequate number of local mental health services. Other barriers were inhospitable behavior of front line mental health staff to potential clients and wait lists to begin services and for individual appointments. One community health worker remarked about the waiting lists, “…when you go to a free clinic…you have to take your kids, you have to pack lunch, you should take sleeping bags, pillows, because you are going to be there a while…” Similar barriers were noted within the agency, such as limited resources and services and overburdened agency staff. Moreover, screening and referral for depression within the agency makes maintaining confidentiality difficult and thus serves as a barrier in providing services. 

### 3.4. Culture and Language

Culture and language emerged in relation to addressing PPD within different languages, cultures and undocumented status. The agency serves a very diverse population which speaks various languages, such as Spanish, French or French Creole. As a result of this diversity, there are cultural norms that make addressing PPD difficult. For example, there may be a reluctance to allow the community health worker in the home because of past experiences or cultural taboos. Some clients are also reluctant to discuss mental health concerns because they feel these issues should be kept in the family. Additionally, undocumented immigrants may not have insurance or financial means to access services and many are reluctant to seek services because of their fear of the system. One community health worker stated, “we deal with a lot of undocumented immigrants (and) a lot of people with different cultural diversities…(allowing)…strangers…into their home or even discussing certain things over the telephone is difficult and sometimes just taboo for some cultures…So that is a roadblock that we constantly come upon.” Cultural issues can also affect communication between the community health worker and the client. A worker shared a story about a client joking around about her life insurance and a supervisor took this quite literally and reacted as if the client might harm herself, which offended the client.

### 3.5. Life Events

Community health workers expressed difficultly differentiating between PPD and the life events challenging their clients. The women have economic, social, educational and living challenges that are numerous and appear to be masked as depression. Community health workers state that many clients screen at risk for PPD because of their economic, educational or interpersonal situations and are not really at risk for or suffering from depression but have other needs that require assistance. Other clients may actually be suffering from depression but the negative life events can be so overwhelming that clients believe there are more pressing concerns than their depressive symptoms or seeking services

“I have this one client…(who) has so many issues going on, abusive relationship which she got out of and then custody battle with the children that are going to be a year in June and she is also pregnant…She had so much going on that she rejected the (mental health) referral. She was there in my office when we called in and set up an appointment for her to go because she is deeply depressed and she rejected it because…she…told me…and…the intake worker, “I just have so much going on, I have to be in court,…I do not have time to sit here on the phone or sit in your office and actually do an assessment regarding my mental health issues. I have my family to take care of…”.

### 3.6. Support

Support encompasses both agency support and family support. Agency support includes monitoring client progress, providing inspiration, developing a trusting relationship, collaboration and providing a sense of normalcy. Community health workers state that they are monitoring clients' PPD symptoms by checking in with them on a regular basis and checking in with supervisors. They share what they term “inspirational stories” by others who have succeeded to help motivate clients and try and induce them to take ownership of their own mental health. Gaining trust and building relationships takes time. Workers also perceive themselves as providing general support and reducing clients' isolation. 

Another important support function is normalizing symptoms and situations. Many new mothers may not know how to interpret their feelings, thoughts and behaviors and community health workers assist them in explaining and understanding their symptoms. As one worker commented

“One other thing I really try to do is, especially if I know the client is depressed,…is to encourage her that this is just a normal process after having a baby…You are not crazy…Give yourself a week or so and if you find that you are not coming around then maybe we need to start talking about taking the next step, as long as you do not feel like you want to hurt the baby and you are able to take care of the baby…It's okay not to be…jumping around with joy…I always tell them that, it…give(s) them a sense of peace…”

Family members can greatly impact the women in both supportive and nonsupportive ways. At times, family members are viewed as part of the team. Other times clients may not want their families to know they're receiving services or the community health workers may be perceived as bringing trouble into the home. The presence of family members can impose on the confidentiality of the screening if the family member (usually the spouse or partner) tries to influence responses to the questions. As one community health worker commented, “I think that is a difficult time when you are dealing with certain cultures (or)…the culture in their house, when the man is…always present (and) has that strong voice for the client and you just cannot separate them. Even on the phone, you can hear his voice in the background.”

## 4. Discussion

The purpose of this study was to obtain qualitative data to inform the practice of community-based screening and mental health referral for postpartum women with depression. Focus groups were conducted with community health workers to understand the barriers and facilitators to refer postpartum women to mental health services and to document their current screening and referral practices. The focus groups revealed that the community health workers address numerous issues in screening and implement a variety of strategies to assist a postpartum woman into accessing mental health services. 

The focus group discussion illustrated that the community health workers viewed screening and referral for PPD as an important aspect of their work. Training was pivotal in their preparation for these tasks. The focus group findings indicate that continuous training and education about screening, as well as referral, are needed. Community health workers suggested some skepticism about the screening tool. In another qualitative study, health care providers in Canada stated that current assessment tools were inadequate for screening PPD in immigrant women [29]. Furthermore, the workers find it difficult to distinguish between a depressive disorder and a normal reaction to a stressful life event. These issues indicate the need for further training about PPD, its detection and the screening tools. 

The barriers identified by the community health workers are similar to those described in the research literature. A variety of personal, practical, and organizational barriers hinder postpartum mothers from utilizing available mental health services. Barriers to mental health services for urban, low-income postpartum women include mental health stigma, racial discrimination in the health care system, lack of social support, costs, transportation, and competing demands of parenting/care-giving [20, 30, 31]. There may be cultural barriers if the postpartum women are from immigrant or ethnic minority populations [29, 32]. Many of these barriers can be directly addressed and attention to them may improve effective referral to mental health services.

Several facilitators were described by the community health workers. They developed trusting, supportive relationships with the women and helped address personal and logistical barriers. The workers encouraged the women to seek services but also helped arrange the initial mental health appointment and transportation. At times, the community health workers accompanied their clients to the appointment. Similarly, community health workers developed a collaborative relationship with mental health agencies to get their clients the appropriate services. In a qualitative study of postpartum women referred for services, participants identified trusting relationships with health providers, outreach, and encouragement to seek services as facilitators to mental health service receipt [33]. Another qualitative study of pregnant, low-income women demonstrated that ways to overcome barriers to seeking mental health services included facilitating trust between women and their providers, education about the consequences of not getting help, being aware of resources and identifying depressive symptoms [34]. Our findings are consistent with these other qualitative studies and together indicate concrete ways in which mental health referral services can be enhanced. 

Clearly, there are intervention strategies that can be utilized to assist postpartum women with depression to engage in mental health services. A nurse-delivered intervention for depression included several steps, such as notifying physicians of their perinatal patients' positive screening score, giving depression feedback to patients and referring patients to treatment [35]. The feedback included education about depression and the referral included an on-site social worker whose first appointment was offered for no fee for uninsured women. Approximately 30% of women reported using depression treatment after the intervention and up to six weeks postpartum, however, only 10% of women were being treated three months prior to their initial prenatal visit, indicating that the intervention improved mental health utilization. Additionally, recommendations for this population include providing several referral options to allow for choice, as well as referrals that are timely and convenient. The utilization of multiple strategies appears optimal [1]. 

There are limitations to the study. The sample size was small, although it represented about half of the community health workers from the partnership agency. It is likely that the community health workers who were more interested in mental health issues and competent with referral were more likely to attend the focus groups and thus the sample may be a biased sample. Additional focus groups with postpartum mothers would have strengthened the current study. 

Despite these limitations, the preliminary findings suggest several future research directions. Future research can employ qualitative interviews and/or surveys with mental health agencies to gain understanding of their policies and practices that impact the services provided to postpartum women. The referral process to mental health services is complex and can to be examined in multiple ways. An additional research direction is to empirically test the facilitators (e.g., arranging transportation, assisting in making the first call to the mental health agency) by the community health workers to see if they increase a postpartum women's uptake of mental health services including further assessment. Finally, the community health workers expressed concerns about potential negative consequences of screening. In order to examine this, more research is needed on the screening process for postpartum depression to determine its impact on women's general functioning and consequent health care receipt. 

## 5. Conclusion

Clearly, community health workers can be actively engaged in screening for PPD and referral to mental health services, especially for low-income women. Universal screening for PPD can be taxing on a health care system and the use of paraprofessionals may be a way to alleviate the burden and improve access, especially for underserved populations. Referral to mental health services for depression care is a complex issue with multiple layers of interactions among systems. We recommend the development of a manualized set of referral strategies to facilitate access to care for depression based on identification of common barriers and effective solutions, as well as procedures for systematic followup to initial referral efforts. 

## Figures and Tables

**Table 1:** Sample characteristics of focus group participants.

	*N*	%
Number of years worked at the agency		
Less than 1 year	4	25.0
1 to 3 Years	6	37.5
More than 3 years	6	37.5
Positions held		
Community health workers	13	81.2
Managers	3	18.8
Years of education		
High school graduates or GED equivalency	1	6.2
Some college	3	18.8
Associates degree	1	6.2
College degree	5	31.2
Some graduate training or degree	4	25.0
Other	2	12.5
Racial/ethnic composition		
African American	8	50.0
Latino	4	25.0
White	3	18.8
Asian	1	6.2
